# Linking Empowering Leadership to Task Performance, Taking Charge, and Voice: The Mediating Role of Feedback-Seeking

**DOI:** 10.3389/fpsyg.2018.02025

**Published:** 2018-10-25

**Authors:** Jing Qian, Baihe Song, Zhuyun Jin, Bin Wang, Hao Chen

**Affiliations:** ^1^Department of Human Resource Management, Business School, Beijing Normal University, Beijing, China; ^2^Faculty of Basic Medical Science, Kunming Medical University, Kunming, China; ^3^Future of Work Institute, Curtin University, Perth, WA, Australia

**Keywords:** empowering leadership, feedback-seeking, task performance, taking charge, voice

## Abstract

Drawing upon social exchange theory, the present study focuses on the role of feedback-seeking in linking empowering leadership to task performance, taking charge, and voice. We tested the hypothesized model using data from a sample of 32 supervisors and 197 their immediate subordinates. Performing CFA, SEM, and bootstrapping, the results revealed that: (1) empowering leadership was positively associated with followers’ feedback-seeking; (2) employees’ feedback-seeking was positively correlated with task performance, taking charge, and voice; and (3) employees’ feedback-seeking mediated the positive relationships between empowering leadership and task performance, taking charge, and voice. We make conclusions by discussing the theoretical and practical implications of these findings, alongside a discussion of the present limitations and directions for future studies.

## Introduction

Currently, the accelerating development of knowledge-based economies brings about uncertainty, changes, and dynamic conditions within organizations (Detert and Burris, 2007; Parker and Collins, 2010; Martin et al., 2013; Li et al., 2017). Empowering leadership, as an effective leadership style enabling organizations to efficiently deal with complex situations, has been increasingly emphasized (Lee et al., in press). This type of leadership consists of supervisors enhancing subordinates’ motivation and generating self-efficacy and psychological empowerment by sharing power with or granting more autonomy to their followers (Kirkman and Rosen, 1999; Arnold et al., 2000; Li et al., 2015, 2017). The positive outcomes of empowering leadership include creativity (Amundsen and Martinsen, 2015), citizenship behavior (Li et al., 2017), in-role performance (i.e., performance that is formally expected of subordinates; Kim and Beehr, 2017b), job satisfaction (Fong and Snape, 2015), and career commitment (Kim and Beehr, 2017a). Recent work (Li et al., 2015; Hao et al., 2017; Lee et al., in press) has further suggested that empowering leadership is positively related to task performance (i.e., a particular aspect of an employee’s in-role performance) (Hao et al., 2017), taking charge (Li et al., 2015), and voice (Yoon, 2012). We contribute to this important line of research by developing and investigating a model that explains how and why empowering leadership is positively related with task performance, taking charge, and voice.

A handful of studies have investigated the psychological mechanisms behind the influencing process of empowering leadership, such as self-efficacy and psychological ownership (Kim and Beehr, 2017b), psychological empowerment (e.g., Raub and Robert, 2010; Auh et al., 2014; Amundsen and Martinsen, 2015), role breadth self-efficacy (e.g., Li et al., 2015), and passion for work (e.g., Hao et al., 2017); however, very little research has been conducted from the behavioral perspective. In light of this, the present study explores an important proactive behavior, that is, employees’ feedback-seeking behavior, mediating between empowering leadership and an in-role outcome (i.e., employees’ task performance), as well as two extra-role outcomes (i.e., employees’ taking charge and voice). Previous studies have made progress in exploring the relationship between psychological empowerment and feedback seeking behavior (Chen et al., 2007; Huang, 2012). For example, Huang (2012) suggests psychological empowerment is positively associated with feedback-seeking behavior mediated by trust in one’s immediate supervisor; and Chen et al. (2007) demonstrates the relationship of LMX and negative feedback-seeking behavior is negatively moderated by subordinates’ own sense of empowerment which is positively related to a team’s empowerment climate. However, the constructs of empowering leadership and psychological empowerment are definitely different. Empowering leadership refers to empowering leaders’ certain behaviors, such as sharing power with employees, which can be perceived by subordinates (Kirkman and Rosen, 1999; Arnold et al., 2000; Li et al., 2015, 2017); while psychological empowerment is defined as employees’ intrinsic motivational construct (Huang, 2012). Despite existing researches that illustrates the effects of both psychological empowerment on feedback seeking behavior and empowering leadership on psychological empowerment (Chen et al., 2007; Raub and Robert, 2010; Huang, 2012; Auh et al., 2014; Amundsen and Martinsen, 2015), the relation between empowering leadership and feedback seeking behavior is still unknown. This study addresses this gap by examining feedback seeking behavior as a mediator. We also extend the literatures investigating on psychological mechanisms behind the influencing process of empowering leadership by applying a new lens of behavioral perspective.

Scholars suggest that feedback-seeking behavior is a particular type of proactive behavior (i.e., proactive person-environment fit behavior), which refers to proactive behaviors that focus on changing oneself to gain better compatibility (Parker and Collins, 2010). It is especially relevant for proactive performance improvement (Huang, 2012). Via feedback-seeking behavior, employees can better respond to the requirements of situations and therefore behave more effectively within organizations (Parker and Collins, 2010). Indeed, there is a longstanding view that feedback-seeking behavior is an important proactive strategy in employees’ adaptive processes (Ashford, 1986; Parker and Collins, 2010). In this study, we apply social exchange theory (Blau, 1964; Emerson, 1976) to explain the mediating role of feedback seeking in the relationships between empowering leadership and work outcomes. Social exchange theory suggests that high-quality social exchange relationships obey the norm of reciprocity (Blau, 1964; Emerson, 1976). More concretely, the recipients of benefits are somehow obligated to provide returns to the givers (Emerson, 1976). Feedback-seeking behavior is considered to be an important behavioral strategy, enabling individuals to enhance their abilities, thus can repay empowering leaders’ benefits (Harris et al., 2014; Parker and Collins, 2010). Feedback-seeking helps a person improve his or her performance and brings about desirable outcomes (Huang, 2012; Ashford et al., 2016), such as improved task performance (Chen et al., 2007), voice behavior, and taking charge. Taken together, we consider employees’ feedback-seeking behavior as a potential mediator and argue that empowering leadership could foster employees’ feedback-seeking behavior and in turn promote both employees’ in-role performance (i.e., task performance) and extra-role performance (i.e., taking charge and voice).

**FIGURE 1:**
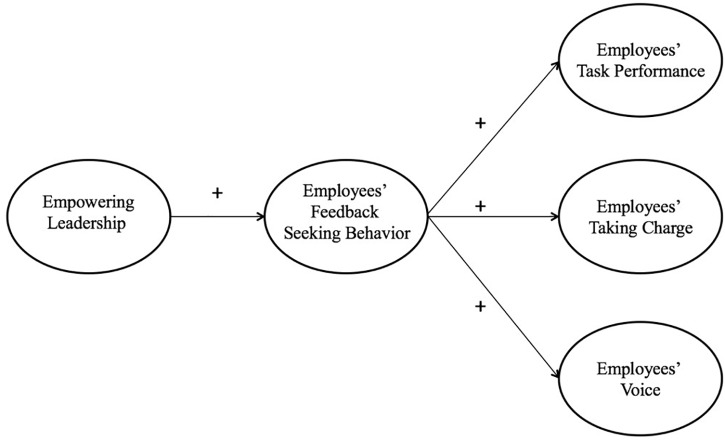
Theoretical model.

Thus, the first contribution of the current study is to extend our understanding of the relationship between empowering leadership and employee task performance by examining employees’ feedback-seeking behavior as a mediator of this relationship (Whitaker et al., 2007; Nifadkar et al., 2012; Hao et al., 2017; Lee et al., in press). Our second contribution is to advance the integration of multiple proactive behaviors (i.e., taking charge, voice, and feedback-seeking behavior; Parker and Collins, 2010), and the ongoing research stream of identifying the outcomes of feedback-seeking behavior (Ashford et al., 2016). Our third contribution is to provide empirical evidence for the relationship between empowering leadership and feedback-seeking behavior. The hypothesized theoretical model is presented in Figure 1.

## Theory and Hypotheses

### Empowering Leadership and Feedback-Seeking

Empowering leadership can provide strong support for employees via a series of positive managerial practices, such as encouragement, emotional support, and information giving (Fong and Snape, 2015; Li et al., 2015). Understandably, empowering leadership is demonstrated to have positive influences on subordinates’ proactive behaviors (e.g., Zhang and Bartol, 2010; Chen et al., 2011). One critical proactive feedback-seeking behavior is commonly explained to be antecedent-orientated in accordance with three basic motives (i.e., instrumental, ego, and image, Ashford et al., 2003, 2016). In the present study, we suggest that empowering leadership will stimulate followers’ feedback-seeking behaviors by increasing its instrumental value as well as decreasing the ego and image costs of feedback.

Specifically, empowering leadership focuses on sharing power and autonomy (Harris et al., 2014). Empowering leaders often delegate power and autonomy to their followers through affirming the importance of subordinates’ work, showing confidence in subordinates’ abilities, and transferring information and resources, as well as offering more opportunities of autonomous decision-making and problem-solving (Martin et al., 2013; Li et al., 2015; Lee et al., in press). Responding to the support of an empowering leader, an employee may feel loyal to his/her colleagues and feel that the leader’s responsibilities should be shared amongst the workers (Srivastava et al., 2006; Li et al., 2015). The higher layer of responsibility, in turn, may require the employees to demonstrate more advanced abilities and skills in the workplace. As such, employees’ perceptions of the instrumental value of feedback for developing competence may strongly increase. Indeed, previous research has shown that newcomers seek feedback more frequently when their work needs higher levels of skills (Wanberg and Kammeyer-Mueller, 2000; Ashford et al., 2003). More directly, a recent review suggests that subordinates are more willing to engage in feedback-seeking when they are given more autonomy (Ashford et al., 2016).

In addition, having gained greater power and autonomy from the empowering leaders, employees may experience more adaptability and flexibility in organizational contexts (Lee et al., in press). As a result, employees feel better about themselves and their self-confidence of engaging in risky behaviors (such as feedback seeking, voice, and taking charge) is enhanced (Ahearne et al., 2005; Li et al., 2015). In addition, previous research has shown that employees with higher self-confidence are more likely to seek feedback (Ashford, 1986; Ashford et al., 2003), and empowering leadership is capable of reducing subordinates’ potential costs of being proactive (Martin et al., 2013). If an individual has high amounts of self-confidence, they are less inclined to worry about the cost to their image when seeking feedback (Ashford et al., 2003). Therefore, we argue that under the management of empowering leaders, employees may weigh the instrumental benefits of feedback-seeking against the costs of potential ego and image. Accordingly:

Hypothesis 1: Leaders’ empowering leadership is positively related to employees’ feedback-seeking behavior.

### Feedback-Seeking Behavior and Task Performance

Feedback-seeking behavior is positively associated with in-role performance (Nifadkar et al., 2012). Task performance is considered as a particular aspect of an employee’s in-role performance within organizations (Shea and Howell, 1999; Parker and Collins, 2010). Specifically, previous studies propose that when employees want to achieve good work performance, they must develop a precise understanding of their role and task requirements (Renn and Fedor, 2001; Whitaker and Levy, 2012). For the sake of the knowledge about the self and tasks, employees will search for relevant information to the best of their ability (Korman, 2001; Whitaker and Levy, 2012). Feedback-seeking behavior is considered to be instrumental in obtaining such information (Ashford and Cummings, 1983; Ashford, 2003). Not surprisingly, feedback-seeking behavior positively influences several performance outcomes, such as task performance (Lee et al., in press), individual creative performance (Hao et al., 2017), and team creative performance (Hon and Chan, 2013). Indeed, empirical studies have demonstrated that feedback-seeking can exert positive effects on task performance (e.g., Whitaker et al., 2007; Whitaker and Levy, 2012). In the present study, we suggest that by using the performance-related information obtained by feedback-seeking, employees can have a better understanding of the task expectations, as well as how to cover any shortages in order to meet these expectations, which in turn helps them work more efficiently and achieve desirable task performance.

Hypothesis 2: Employees’ feedback-seeking behavior is positively related to their task performance.

### Feedback-Seeking Behavior and Taking Charge

Taking charge is defined as an extra-role behavior reflecting one’s voluntary and constructive efforts to challenge the status quo and bring about organizational functional change (Morrison and Phelps, 1999). Taking charge is beneficial for organizational effectiveness (Morrison and Phelps, 1999); however, employees’ taking-charge behavior is usually withdrawn. Taking charge is characterized as risky (McAllister et al., 2007; Li et al., 2015). If an individual’s proposal is seen as inappropriate or threatening, the individual’s reputation in the workplace will be damaged (Morrison and Phelps, 1999). A previous study notes that challenging the status quo, which is one of the important aims of taking charge, is likely to annoy the leaders and generate negative career consequences (Detert and Edmondson, 2011). Morrison and Phelps (1999) suggest that two key judgments determining the decision to take charge are assessments of likely success and likely consequences. We suggest that feedback seeking can enhance employees’ assessments of the probability of success and reduce their assessments of potential risks with regard to taking charge. Specifically, frequent feedback-seeking allows employees to acquire information that helps them identify work-related problems accurately and function productively (Ashford et al., 2003, 2016). This can improve their possibility of bringing about organizational functional change successfully. As such, employees are likely to underestimate the potential risks and believe they are more likely to be successful if they take charge (Morrison and Phelps, 1999). Accordingly:

Hypothesis 3: Employees’ feedback-seeking behavior is positively related to their taking charge.

### Feedback-Seeking Behavior and Voice

Defined as one’s communication of constructive opinions, concerns, or suggestions about problems or other work-related issues, voice is also seen as an extra-role behavior aiming to improve or change organizations (van Dyne et al., 2003; Morrison, 2011). Similar to taking charge, voice behavior is characterized as risky because it often challenges authority and reveals negative aspects that others avoid mentioning (Detert and Burris, 2007; Venkataramani and Tangirala, 2010; Morrison, 2011; Maynes and Podsakoff, 2014). Before making the decision to carry out voice behavior, employees should not only have the ability to notice the potential problems (i.e., perceived efficacy of voice) but also have the confidence in their ability to speak up about the problems (i.e., perceived safety of voice) (Morrison, 2011, 2014). We argue that feedback-seeking benefits these conditions and in turn stimulates employees’ voice behavior. This is because feedback-seeking can help employees build all-round communication channels through which they can access solid and comprehensive information resources, such as knowledge, material, and expertise sharing between peers (Ashford, 1986; Ashford et al., 2003, 2016; Anseel et al., 2015). Accessing these resources means that employees see things from a more comprehensive perspective. They are likely to have greater opportunity to discover upcoming problems or inefficient or inappropriate activities and subsequently come up with solutions (Morrison, 2014). They can feel greater personal control over voice. As a result, their perceived self-efficacy and safety of voice may increase. There is evidence that feedback-seeking may improve a person’s self-efficacy when he or she deems that feedback-seeking can bring about positive performance (Renn and Fedor, 2001). Accordingly:

Hypothesis 4: Employees’ feedback-seeking behavior is positively related to their voice.

### The Mediating Roles of Feedback-Seeking Behavior

In the present study, drawing on social exchange theory (Blau, 1964; Emerson, 1976), we argue that employees are likely to develop high-quality social exchange relationships with the leaders under the management of empowering leaders. Specifically, we suggest that through a series of positive managerial practices such as encouragement, emotional support, and information giving, empowering leaders can enhance their followers’ perceptions about the quality of their relationship, personal influence, and power in the workplace, or psychological safety. According to social exchange theory (Blau, 1964; Emerson, 1976), as a way to reciprocate these benefits offered by the leaders, subordinates may feel more motivated to meet supervisors’ demands rather than feel stress that results with them shrinking from those demands. Feedback-seeking behavior gathers the necessary information and increases the likelihood of success for employees promoting their performance to meet supervisors’ demands (Ashford et al., 2016). Given that the new demands include shared responsibility for the supervisors, employees may have to improve their abilities to behave in the context of a team. Specifically, to reciprocate empowering leaders’ benefits, subordinates are supposed to not only improve their in-role performance (i.e., task performance in this study) but also sharpen their extra-role performance by voluntarily effecting organizationally functional change (i.e., taking charge) or by proactively communicating ideas, suggestions, and so on (i.e., voice). Accordingly:

Hypothesis 5a: Employees’ feedback-seeking behavior mediates the relationship between leaders’ empowering leadership and employees’ task performance.
Hypothesis 5b: Employees’ feedback-seeking behavior mediates the relationship between leaders’ empowering leadership and employees’ taking charge.
Hypothesis 5c: Employees’ feedback-seeking behavior mediates the relationship between leaders’ empowering leadership and employees’ voice.

## Materials and Methods

### Participants and Procedure

We collected the data from a logistics company located in northern China. This survey involved 224 employees and their immediate supervisors from 32 workgroups. We conducted the survey with the support of the company’s human resources department. Participants voluntarily participated in this survey without receiving any specific rewards. Participants’ written informed consent was obtained before the distribution of questionnaires. We prepared separate questionnaires for supervisors and subordinates to minimize the common method bias; supervisor participants and subordinate participants completed their questionnaires, respectively. Identification numbers were used to match subordinates’ responses with their immediate supervisors’ responses. To ensure confidentiality, we provided a return envelope with seal tape for each respondent. We sent two e-mails to remind each employee to seal the finished questionnaire in the envelope and to return it at a company-wide meeting 2 weeks later. At the meeting, one of the researchers placed a secure box outside the venue and instructed the participants to put their sealed questionnaires into the designated box. All these procedures were conducted in accordance with the ethical standards of the institutional and/or national research committee and with the 1964 Declaration of Helsinki and its later amendments or comparable ethical standards with written informed consent from all subjects. The present study was approved by the Human Research Ethics Committee (HREC) at the Business School of Beijing Normal University. As a result, 32 supervisor questionnaires and 197 subordinate questionnaires were returned (i.e., 100 and 87.9% response rate, respectively), which composed the final sample. The final samples of 197 subordinate respondents were predominantly male (63.5%, *SD* = 0.48). Most of them held bachelor degrees (34.5%), 18.8% held junior college degrees, 21.3% held senior high school degrees, and the rest held junior high school degrees (23.9%). The average age of the participants was 29.13 years (*SD* = 5.28). Rates of missing data ranged from 0 to 0.5%; all missing data were due to participant non-response (e.g., deliberately or accidentally not responding to certain items).

### Measures

All survey instruments were originally constructed in English. Following Brislin (1980), we translated them into Chinese by performing a standard translation and back-translation procedure.

#### Empowering Leadership

Empowering leadership was measured using the 12-item scale developed by Ahearne et al. (2005). Response options ranged from 1, “strongly disagree” to 7, “strongly agree”. An example item is, “My leader believes that I can handle demanding tasks.” (Coefficient alpha = 0.86).

#### Feedback-Seeking Behavior

Feedback-seeking behavior was measured using the 5-item scale developed by VandeWalle et al. (2000). Response options ranged from 1, “never” to 7, “always.” An example item is, “How often does this subordinate ask you for feedback about his or her overall job performance?” (Coefficient alpha = 0.89).

#### Task Performance

Task performance was measured using the 7-item scale developed by Williams and Anderson (1991). Response options ranged from 1, “strongly disagree” to 7, “strongly agree.” An example item is, “This subordinate performs tasks that are expected of him/her.” (Coefficient alpha = 0.88).

#### Taking Charge

Taking charge was measured using the 10-item scale developed by Morrison and Phelps (1999). Response options ranged from 1, “strongly disagree” to 7, “strongly agree.” An example item is, “This subordinate often tries to correct a faulty procedure or practice.” (Coefficient alpha = 0.86).

#### Voice

Voice was measured using the 6-item scale developed by LePine and Van Dyne (1998). Response options ranged from 1, “almost never” to 7, “always.” An example item is, “This subordinate speaks up and encourages others in this group to get involved in issues that affect the group.” (Coefficient alpha = 0.94).

#### Control Variables

Since employees’ behaviors vary according to their individual differences, we included employees’ age, gender, and educational level as potentially confounding variables. Table 2 shows the descriptive statistics and correlations among our study variables and potentially confounding variables. As seen in Table 2, gender and educational level were correlated with some of the outcome variables. Specifically, gender was correlated with taking charge and educational level was correlated with taking charge and task performance. Based on these results, gender and educational level were controlled for in the mediation model.

### Analysis Strategy

First, we performed a confirmatory factor analysis (CFA) to test the adequacy of our measurement model. The assumptions of CFA were specified as recommended by Hau et al. (2004): (1) the mean values of the error terms were 0; (2) there were no correlation between error terms and factors; and (3) the error terms in the measurement equations were not related to each other. Next, structural equation modeling (SEM) was performed using MPLUS 7.4 to test the hypothesized mediation model with latent variables. The assumptions of SEM were specified as follows as recommended by Hau et al. (2004): (1) the mean values of the error terms of the measurement equations were 0; (2) the mean value of the residual error of the structural equation was 0; (3) there was no correlation between error terms and factors in the measurement equations, and the error terms in the measurement equations were not related to each other; and (4) there was no correlation between the residual error in the structural equation and the factors and error terms in the measurement equations. The bootstrapping method was used to generate 95% confidence intervals that estimated the size and significance of the indirect effect; this was recommended as a more powerful analysis for the examination of mediation models and more robust to violations of distribution (Shrout and Bolger, 2002).

Previous literatures argue that fairly large samples are needed both at individual and group levels to conduct multi-level analyses (Hox, 1998). For example, Kreft (1996, Unpublished) suggests that the samples should consist of more than 30 groups, with more than 30 individuals in each group. Hox (1998) suggests the 50/20 rule (more than 50 groups with at least 20 individuals per group) and the 100/10 rule (more than 100 groups with at least 10 individuals per group). Considering that our sample size did not meet these criteria, one level of statistical analysis was adopted in this study.

## Results

### Confirmatory Factory Analysis

As seen in Table 1, our proposed 5-factor measurement model had an acceptable fit (χ^2^ = 683.36, *p* < 0.001, *df* = 467, CFI = 0.96, TLI = 0.95, RMSEA = 0.05, SRMR = 0.07; Hu and Bentler, 1999) and it was better than alternative measurement models. The alternative 3-factor measurement model combined the three outcome variables, and the fit indices were worse than those of our proposed model (χ^2^ = 918.28, *p* < 0.001, *df* = 474, CFI = 0.91, TLI = 0.89, RMSEA = 0.07, SRMR = 0.07). Finally, we combined all variables and had all items load on one factor; again, the fit indices were worse than those of our proposed model (χ^2^ = 1639.31, *p* < 0.001, *df* = 477, CFI = 0.76, TLI = 0.72, RMSEA = 0.11, SRMR = 0.13). Measurement models were re-specified based on modification indices to meet currently accepted criteria.

**Table 1:** Results of confirmatory factor analyses.

	χ^2^	*df*	Δχ^2^	Δ*df*	CFI	TLI	RMSEA	SRMR
Five-factor model (hypothesized model)	683.36	467	–	–	0.96	0.95	0.05	0.07
Three-factor model (combined task performance, taking charge, and voice)	918.28	474	234.92^∗∗^	7	0.91	0.89	0.07	0.07
One-factor model (combined all factors)	1639.31	477	955.95^∗∗^	10	0.76	0.72	0.11	0.13

*Values of Δχ2 were differences of each of the alternative models with the hypothesized five factor model. ^∗^p < 0.05, ^∗∗^p < 0.01.*

**Table 2:** Means, standard deviations, reliabilities, and correlations among study variables.

	Mean	*SD*	1	2	3	4	5	6	7	8
(1) Gender	1.37	0.48	–							
(2) Age	29.14	5.38	−0.01	–						
(3) Educational level	2.69	1.22	−0.39^∗∗^	−0.01	–					
(4) Empowering leadership	5.28	0.79	−0.07	−0.18^∗^	−0.01	(0.86)				
(5) Feedback seeking	4.35	1.01	0.08	0.01	−0.09	0.20^∗∗^	(0.89)			
(6) Taking charge	4.66	0.85	−0.17^∗^	−0.00	0.41^∗∗^	0.14^∗^	0.32^∗∗^	(0.86)		
(7) Voice	4.69	0.87	−0.11	0.07	0.09	0.14^∗^	0.47^∗∗^	0.65^∗∗^	(0.94)	
(8) Task performance	5.05	0.84	−0.17^∗^	−0.00	0.23^∗∗^	0.19^∗∗^	0.42^∗∗^	0.70^∗∗^	0.66^∗∗^	(0.88)

*N = 197. Reliabilities (Cronbach’s α) on the diagonal in parentheses. ^∗^p < 0.05, ^∗∗^p < 0.01.*

### Hypothesis Testing

After establishing the adequate fit of our measurement model, we tested our hypotheses using SEM. The fit indices of the hypothesized model were acceptable (χ^2^ = 821.25, *p* < 0.001, *df* = 532, CFI = 0.94, TLI = 0.93, RMSEA = 0.05, SRMR = 0.08); see Tables 3, 4 for the results and standardized path coefficients of the SEM analyses; see Table 5 for the statistical power of the paths in the SEM model.

**Table 3:** Standardized direct path coefficients of the hypothesized model.

Hypotheses	Paths	Estimate	*SE*
H1	Empowering leadership → feedback seeking	0.34^∗^	0.15
H2	Feedback seeking → task performance	0.43^∗∗^	0.11
H3	Feedback seeking → taking charge	0.41^∗∗^	0.11
H4	Feedback seeking → voice	0.53^∗∗^	0.14
H5a	Empowering leadership → task performance	0.07	0.12
H5b	Empowering leadership → taking charge	0.09	0.13
H5c	Empowering leadership → voice	0.03	0.11

*^∗^p < 0.05, ^∗∗^p < 0.01.*

**Table 4:** Standardized indirect path coefficients of the hypothesized model.

		Bootstrapping		BC 95% CI	
Hypotheses	Paths	Estimate	*SE*	Lower	Upper
H5a	Empowering leadership → feedback seeking → task performance	0.11^∗∗^	0.04	0.03	0.19
H5b	Empowering leadership → feedback seeking → tasking charge	0.10^∗∗^	0.03	0.03	0.17
H5c	Empowering leadership → feedback seeking → voice	0.14^∗∗^	0.05	0.04	0.23

*^∗^p < 0.05, ^∗∗^p < 0.01.*

**Table 5:** The statistical power of the paths in the hypothesized model.

Paths	*R*^2^	Alpha	Power
Empowering leadership → feedback seeking	0.06	0.05	0.95
Feedback seeking → task performance	0.32	0.05	1
Feedback seeking → taking charge	0.34	0.05	1
Feedback seeking → voice	0.23	0.05	1

The first hypothesis predicted that leaders’ empowering leadership was positively related to employees’ feedback-seeking behavior. This hypothesis was supported (β = 0.34, *p* < 0.05).

Hypotheses 2, 3, and 4 predicted that employees’ feedback-seeking behavior would be positively related to their task performance, taking charge, and voice. These effects were found to be significant, for task performance β = 0.43, *p* < 0.01, for taking charge β = 0.41, *p* < 0.01, and for voice β = 0.53, *p* < 0.01. Hypotheses 2, 3, and 4 were supported.

For the mediation hypothesis, we found a significant mediation effect for employees’ feedback-seeking behavior on the relationship between empowering leadership and the outcome variables. H5a predicted that employees’ feedback-seeking behavior mediates the relationship between leaders’ empowering leadership and employees’ task performance, the bootstrapping results indicated that this indirect effect between employees’ feedback-seeking behavior and task performance was significant (β = 0.13, *p* < 0.01; bootstrap bias-corrected 95% CI [0.02, 0.21]). H5b predicted that employees’ feedback-seeking behavior mediates the relationship between leaders’ empowering leadership and employees’ taking charge; the bootstrapping results indicated that this indirect effect between employees’ feedback-seeking behavior and taking charge was significant (β = 0.10, *p* < 0.01; bootstrap bias-corrected 95% CI [0.03, 0.17]). H5c predicted that employees’ feedback-seeking behavior mediates the relationship between leaders’ empowering leadership and employees’ voice; the bootstrapping results indicated that this indirect effect between employees’ feedback-seeking behavior and voice was significant (β = 0.14, *p* < 0.01; bootstrap bias-corrected 95% CI [0.05, 0.22]). H5a, H5b, and H5c were therefore supported.

## Discussion

The present study examines the potential consequences of empowering leadership on subordinates’ in-role performance (i.e., task performance) and extra-role performance (i.e., proactive behaviors such as feedback-seeking, voice, and taking charge). The results support our hypotheses, revealing that: (1) empowering leadership positively relates to feedback-seeking behavior; (2) feedback-seeking behavior positively relates to task performance, taking charge, and voice; and (3) feedback-seeking behavior mediates the relationships between empowering leadership and task performance, taking charge, and voice.

### Theoretical Implications

Our findings offer several theoretical contributions to the empowering leadership and feedback-seeking literatures. First, our findings concerning the relationship between empowering leadership and feedback-seeking extends the research stream of identifying feedback-seeking’s antecedents by investigating empowering leadership as a predictor (e.g., Barner-Rasmussen, 2003; Huang, 2012; Qian et al., 2012; Chun et al., 2014; Anseel et al., 2015). Previous studies have emphasized the importance of leaders encouraging the feedback-seeking behavior subordinates, such as authentic leadership (Qian et al., 2012, 2016) and transformational leadership (Anseel et al., 2015). Although scholars have attached importance to supervisors’ influences on followers’ feedback-seeking behaviors, little is known about the relationship between empowering leadership and feedback-seeking. Our findings fill this gap and show that employees are more motivated to engage in feedback-seeking behavior under the management of empowering leaders.

Second, our findings note that employees’ feedback-seeking behaviors can enhance their task performance, taking charge, and voice. Previous studies have shown that employees who frequently seek feedback gain better task performance (Whitaker et al., 2007). Our findings advance Whitaker et al. (2007) work by revealing that feedback-seeking behavior cannot only improve employees’ in-role performance (i.e., task performance) but also enhance their extra-role performance (i.e., taking charge and voice). Our findings concerning the relationship between feedback-seeking and taking charge and voice also extends current knowledge of the consequences of feedback-seeking (Whitaker and Levy, 2012; Ashford et al., 2016; Gong et al., 2017). Additionally, previous scholars have identified three types of proactive behaviors and call for future researchers to investigate the relationships between different proactive behaviors (Parker and Collins, 2010). As a response to Parker and Collins (2010) call, our finding suggests that feedback-seeking behavior, as a proactive person-environment fit behavior, enhances the two proactive work behaviors, i.e., taking charge and voice. This finding contributes to the integration of proactive behaviors (Parker and Collins, 2010).

Third, our findings demonstrate that feedback-seeking behavior fully mediates the relationships between empowering leadership and task performance, taking charge, and voice. Though previous studies have demonstrated that empowering leadership is associated with voice or taking charge (Yoon, 2012; Li et al., 2015). Indeed, prior findings with regard to full or partial mediating roles in the relationship between empowering leadership and extra-role behavior is contradictory (e.g., Raub and Robert, 2010; Yoon, 2012). For example, Raub and Robert (2010) found that psychological empowerment fully mediates the relationship between empowering leadership and challenging extra-role behaviors. In Yoon (2012) paper, however, the relationship of empowering leadership and voice behavior is partially mediated by psychological empowerment. In the present paper, we suggest that leaders’ empowering behaviors may give employees reasons to voice or taking charge, given that empowering leaders are likely to develop high-quality social exchange relationships with followers (Blau, 1964). That is why previous scholars identify the direct relationships between empowering leadership, voice, and taking charge (Yoon, 2012; Li et al., 2015). However, just having reasons is not enough when employees engage in risky behaviors (McAllister et al., 2007; Morrison, 2011; Li et al., 2015). Employees must have ability and confidence to engage in these extra-role behaviors. Feedback-seeking behaviors helps them gain work-related information (Ashford et al., 2003, 2016), thus giving employees ability and confidence to voice and taking charge. Although Baron and Kenny (1986) suggest that full mediation is the most powerful proof of the existence of a mediating effect, the distinction between complete and partial mediation is only one of the ways of verbal descriptions of the effect size of the mediational models (Preacher and Kelley, 2011). In fact, this does not mean that direct effects must not exist in fact. Actually, Preacher and Kelley (2011) argued that the notion of full mediation should be abandoned and all mediations be treated as partial mediations. Thus, we should interpret the results of this mediational model with caution.

Fourth, scholars began to emphasize the importance of examining feedback-seeking as a critical mediating mechanism (Ashford et al., 2016). According to social exchange theory (Blau, 1964; Emerson, 1976), we argue that employees use feedback-seeking as an adaptive strategy to reciprocate empowering leaders’ benefits, which in turn enhances their task performance, taking charge, and voice. By using social exchange theory, this study provides a new theoretical lens for understanding the mediating roles of feedback seeking.

### Practical Implications

Our findings offer several implications for the managerial challenges of enhancing employees’ in-role performance (i.e., task performance) and extra-role performance (i.e., feedback-seeking behavior, taking charge, and voice). First, our findings show that empowering leadership plays an important role in stimulating followers’ feedback-seeking behaviors and following positive outcomes of performance enhancement, voice, and taking charge, which provides a new method for managerial practitioners to motivate subordinates to seek feedback and generate positive work outcomes. When recruiting and selecting managers, organizations should pay close attention to the personality traits of candidates in light of recent discoveries in the field of empowering leadership (Li et al., 2015). For example, prior studies argue that individuals who have a high need for achievement tend to fail to empower (Li et al., 2015); while supervisors who possess high levels of humility are very likely to show empowering leadership behaviors (Ou et al., 2014). In terms of training and encouraging managers to be empowering, organizations may require managers to participate in executive education programs or attend leadership centers and introduce empowering leadership behaviors into the performance evaluation system (Amundsen et al., 2014). Second, our findings indicate that feedback-seeking behavior has positive influences on task performance, taking charge, and voice, and mediates the relationships between empowering leadership and these outcomes. Accordingly, this study offers new insights into how to enhance employees’ in-role performance (i.e., task performance) and extra-role performance (i.e., taking charge and voice). In terms of employee recruitment, organizations can take individual differences associated with feedback-seeking behavior into account, such as feedback orientation (Dahling et al., 2012) and emotional intelligence (Kim et al., 2009). Additionally, when performing empowering behaviors to cultivate follower proactivity and performance improvement, supervisors should also take efforts to develop a supportive feedback environment (Dahling et al., 2012; Huang, 2012). For instance, supervisors can consistently provide specific, credible, and high-quality information for effective performance feedback (Dahling et al., 2012).

### Limitations

There are several limitations that require further exploration. First, in the present study we suggested that empowering leadership stimulates followers’ feedback-seeking behaviors, which in turn improves subordinates’ task performance, taking charge, and voice. However, given the cross-sectional nature of this study, we cannot make definitive conclusions of this causality. It is possible that this causal relationship is reversed. For instance, those followers who frequently ask their leaders for feedback are likely to obtain more shared information from their subordinates. The leaders may even appreciate the employees’ proactivity and delegate them more autonomy and power. Future researchers can use longitudinal, experimental, or quasi-experimental designs to address this issue. For example, researchers can collect time-lagged data at several separate points in time (Finkel, 1995; Podsakoff et al., 2003). They may measure empowering leadership, feedback-seeking, and the control variables at Time1 and collect the data for task performance, taking charge, and voice 2 weeks later (i.e., Time2) (Ou et al., 2014). Second, we tested our hypotheses using only using data collected from a single company in a Chinese context, which may limit the generalizability of the present findings. We encourage future scholars to replicate these findings by administrating this survey in other cultures or organizations. Third, we only included the mediating mechanisms in the hypothesized theoretical model without taking potential boundary conditions into consideration. Previous empowering leadership studies placed particular emphasis on examining cultural values (e.g., collectivism and individualism) or individual differences (e.g., power distance orientation and traditionalism) with regard to exploring the influences of empowering leadership on followers’ behaviors (Li et al., 2015; Hao et al., 2017). For example, we suggested that employees with low power distance orientation are more motivated by empowering leaders’ sharing autonomy and power to engage in positive behaviors since those subordinates are less likely to accept an unequal distribution of power (Dorfman and Howell, 1988; Clugston et al., 2000; Li et al., 2015). Fourth, in this paper, we mainly interpret the hypothesized model according to social exchange theory (Blau, 1964; Emerson, 1976). Future studies may provide a new lens by applying other theories. In addition, the present study focuses on behavioral mechanisms to explain the relationship between empowering leadership and outcomes. In future studies, researchers may investigate potential psychological mechanisms and compare the different effects. Finally, we only controlled for participants’ demographic variables (i.e., age, gender, and educational level) in the present study. However, in order to distinguish the predictive effects of empowering leadership from other leaderships, future researchers may control for relevant leadership styles, such as transformational leadership (Ou et al., 2014), and laissez-faire leadership (Wong and Giessner, 2018).

## Conclusion

Based on social exchange theory (Blau, 1964; Emerson, 1976), the present study examines the potential consequences of empowering leadership on employees by investigating feedback-seeking behavior as a mediator. Our findings show that empowering leadership cannot only improve followers’ in-role performance (i.e., task performance) but also enhance subordinates’ extra-role performance (i.e., voice and taking charge) via stimulating employees’ feedback-seeking behavior. Our findings contribute to the ongoing research into empowering leadership and feedback-seeking, as well as the integration of proactive behaviors (i.e., feedback-seeking, taking charge, and voice) (Parker and Collins, 2010). Additionally, our findings provide empirical support and theoretical lens for explicating the mediating roles of feedback-seeking from a social exchange perspective.

## Author Contributions

JQ and BS substantially contributed to the conception and the design of the work as well as the preparation of the draft. BW and HC reviewed it critically and gave important intellectual input. ZJ contributed to the analysis and interpretation of the data.

## Conflict of Interest Statement

The authors declare that the research was conducted in the absence of any commercial or financial relationships that could be construed as a potential conflict of interest. The reviewer SR and handling Editor declared their shared affiliation.
